# Plastic and Heritable Components of Phenotypic Variation in *Nucella lapillus*: An Assessment Using Reciprocal Transplant and Common Garden Experiments

**DOI:** 10.1371/journal.pone.0030289

**Published:** 2012-01-27

**Authors:** Sonia Pascoal, Gary Carvalho, Simon Creer, Jenny Rock, Kei Kawaii, Sonia Mendo, Roger Hughes

**Affiliations:** 1 Molecular Ecology and Fisheries Genetics Laboratory, School of Biological Sciences, Environment Centre Wales, Bangor University, Bangor, Gwynedd, United Kingdom; 2 Department of Biology, Centre for Environmental and Marine Studies (CESAM), University of Aveiro, Aveiro, Portugal; 3 Zoology Department, Centre for Science Communication, University of Otago, Dunedin, New Zealand; 4 Research Center for the Pacific Islands, Kagoshima University, Korimoto, Kagoshima, Japan; University of Canterbury, New Zealand

## Abstract

Assessment of plastic and heritable components of phenotypic variation is crucial for understanding the evolution of adaptive character traits in heterogeneous environments. We assessed the above in relation to adaptive shell morphology of the rocky intertidal snail *Nucella lapillus* by reciprocal transplantation of snails between two shores differing in wave action and rearing snails of the same provenance in a common garden. Results were compared with those reported for similar experiments conducted elsewhere. Microsatellite variation indicated limited gene flow between the populations. Intrinsic growth rate was greater in exposed-site than sheltered-site snails, but the reverse was true of absolute growth rate, suggesting heritable compensation for reduced foraging opportunity at the exposed site. Shell morphology of reciprocal transplants partially converged through plasticity toward that of native snails. Shell morphology of F_2_s in the common garden partially retained characteristics of the P-generation, suggesting genetic control. A maternal effect was revealed by greater resemblance of F_1_s than F_2_s to the P-generation. The observed synergistic effects of plastic, maternal and genetic control of shell-shape may be expected to maximise fitness when environmental characteristics become unpredictable through dispersal.

## Introduction

The relative contributions of plasticity and inheritance to phenotypic expression are crucial quantities for understanding the evolution of adaptive character traits in spatially and temporally variable habitats [1]–[8]. Other things being equal, phenotypic plasticity should be selectively advantageous over local genetic adaptation if progeny are randomly distributed among habitats presenting different fitness requirements [9]. The advantage should be reduced, however, if plasticity only achieves an approximate match to the locally optimal phenotype [10] and/or incurs a significant fitness cost [11]. Nevertheless, co-acting heritable and plastic components of adaptive phenotypic variation have been widely demonstrated in both plants and animals, including intertidal gastropods (e.g. [12]–[18]).

Aquatic snails have become popular models for investigating environmental and heritable components of phenotypic expression because they are easily reared in the laboratory, readily transplanted between habitats in the field, and have measurable phenotypic characters whose variation is closely correlated with physical and biological environmental factors critical to survival [18], [19]. Such factors include water movement, which may dislodge the snail from the substratum [20]–[22], desiccation [23], [24], insolation [25], [26] and predation [27]–[32]. In particular, plastic and heritable components of morphological variation in the dogwhelk *Nucella lapillus* (L.) have been studied in relation to contrasting selection regimes associated with high and low wave exposure [26], [33]–[35]. *N. lapillus* is a predatory snail with limited dispersal ability owing to direct development and a restricted crawling range [36]. The species is commonly found on rocky shores of the North Atlantic, ranging from the most wave-exposed to the most sheltered [37]. Spatial variation in wave-exposure embodies a complex environmental gradient, including amplitude of mechanical forces, temperature variation and risk of desiccation that in turn influence community structure and hence the biological environment experienced by *N. lapillus*. The shell of *N. lapillus* is more globular at sites exposed to wave action and more elongated at sheltered sites [38]. The exposed-site shape incurs less drag [39] and is characterized by a relatively larger, more rounded aperture [24] that accommodates a larger foot. The latter enables stronger attachment to the rock and therefore greater resistance to dislodgement by waves [33], [38]. The elongated sheltered-site shape is associated with slower evaporation by having a relatively smaller aperture [23] and with greater capacity for evaporative cooling through holding a relatively greater volume of extra-corporeal water within the basal whorl [24]. Moreover, the greater internal volume allows snails to withdraw further into the shell [40] and this, together with thickened shell walls and the relatively narrow aperture of the elongated shell [41], hinders attacks by crabs which tend to be abundant at sheltered sites but rare at exposed [28]. Broadly similar adaptive variation in shell morphology has been demonstrated in *Littorina saxatilis* [15], [17], which often co-occurs with *N. lapillus*.

Etter [33] examined variation in relative foot size of *Nucella lapillus* from sites differing in exposure to wave action. Snails from the more exposed site had a relatively larger foot, affording greater adhesion, than those from the more sheltered. Progeny of exposed- and sheltered-site snails developed a similar relative foot size when reared under calm-water conditions in the laboratory, indicating plastic rather than heritable phenotypic variation. Reciprocal transplantation of progeny between sites, however, revealed strong asymmetry in the response to environment. Progeny from the sheltered site transplanted to the exposed came to resemble the native snails by developing a relatively much larger foot (pedal area) than controls replanted on the sheltered site. Progeny transplanted from the exposed to the sheltered site, on the other hand, developed a pedal area only slightly smaller than that of controls. Trussell [22] obtained similar results for *Littorina obtusata*, again finding asymmetric plasticity with sheltered-site snails developing a relatively larger foot in response to exposure to wave action, but not vice versa.

Etter [33] and Trussell [22] interpreted the observed asymmetric plasticity of exposed- and sheltered-site snails as an adaptive response to the risk of error in environmentally cued acclimation [42]. They argued that a reduction in relative pedal area during protracted calm periods on exposed shores would lead to heavy mortality through dislodgement when more typical levels of wave action return. On the other hand, an increase in pedal area during prolonged periods of wave action on sheltered shores would be less likely increase mortality when normal conditions return. Kirby et al. [24], however, demonstrated the adaptive value of slender shell shape, correlated with a relatively smaller foot, under desiccating conditions that must frequently occur on shores sheltered from wave action but exposed to sun or wind. Hence it might equally be predicted that shell shape of the progeny of exposed-site snails should converge toward the sheltered-site phenotype when growing in a sheltered environment.

In contrast to Etter's [33] conclusion that phenotypic plasticity adequately accounts for the difference in relative foot size between exposed and sheltered-site forms of *N. lapillus*, other studies have linked phenotypic variation (shell shape) to genotype [13], [16], [24], [35], [43], [44]. The above genetic studies, however, concerned exposed- and sheltered-site populations also differentiated by karyotype-polymorphism [45]–[47] probably reflecting phylogeographical history [44], which might confound assessment of local adaptation.

To further assess the symmetry of plasticity and heritable variation in adaptive morphology of *N. lapillus*, we performed reciprocal-transplant and common-garden experiments, complemented by population genetic analysis, on an exposed- and a sheltered-site population free of karyotype polymorphism. While substantially corroborating previous studies of *N. lapillus*, our data also reveal new insights on the plastic and heritable components of phenotypic variation of this species.

## Materials and Methods

### Reciprocal transplant experiment

**Figure 1:**
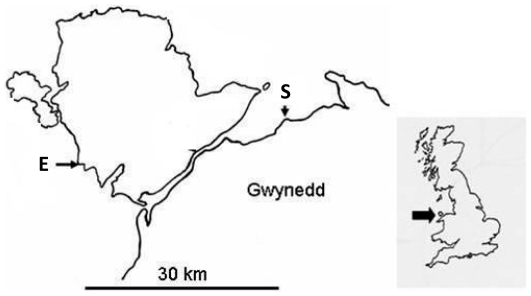
Location of study sites along the North Wales coastline. S = Llanfairfechan (53°15.456′N, 03°58.085′W, exposure index = 1); E = Cable Bay (53°12.410′N, 04°30.290′W, exposure index = 13). The wave exposure index is based on mean annual wind energy and fetch together with environmental modifiers [87].

Two sites in North Wales, UK, were chosen for reciprocal transplantation of *N. lapillus* (Fig. 1). One site, Cable Bay, is exposed to strong wave action generated by prevailing south-westerly winds from the Southwestern Approaches and across the Irish Sea, while the other, Llanfairfechan, is sheltered in the lee of the prevailing winds. The experimental arena at the sheltered site was a glacial boulder of approximately 1.8 m height and 7.2 m circumference. The boulder was covered by a patchwork of barnacles, *Semibalanus balanoides*, and mussels, *Mytilus edulis*, which were densely colonized by barnacles. Near the substratum, the peripheral under-surface of the boulder was bare and was used by adult *N. lapillus* as a refuge. The experimental arena at the exposed site consisted of bed-rock densely populated by the barnacles *Chthamalus montagui* and *Semibalanus balanoides*. The bedrock was devoid of mussels and was dissected by two major crevices used by *N. lapillus* as refuges. One crevice extended 4.5 m along-shore to intersect another running 6 m down-shore. *N. lapillus* foraged within a band some 1.8 m wide along the horizontal crevice and on vertical walls about 1.4 m high either side the down-shore crevice.

#### Laboratory hatchlings

Adult dogwhelks were collected in February 2008 from the exposed and the sheltered site and maintained in site-dedicated aquaria supplied with running seawater closely tracking ambient outdoor temperature. Barnacles were supplied as prey and replenished as needed. The dogwhelks formed spawning aggregations and deposited egg masses on the walls of the aquaria. Once hatched juveniles had grown large enough (8–12 mm, August 2008), they were labelled with a waterproof pen. Labels were covered with superglue (Loctite™) to protect against abrasion and the marked juveniles were released as summarized in Table 1.

**Table 1:** Number of juveniles captured and released per treatment.

Captured	Sheltered		Exposed		Lab sheltered		Lab exposed		Total
**N**	1385		1220		272		400		3277
**Released**	SS	SE	EE	ES	lSS	lSE	lEE	lES	
**N**	514	871	610	610	72*	200	200	200	3277

S: sheltered; E: exposed; f: juveniles collected from the field; l: laboratory-hatched juveniles. Treatment-labels are as in Fig. 4.

*The low number was caused by mortality during marking.

Each of the above treatments was given a unique colour-code. On release, individuals were gently irrigated with seawater to encourage them to emerge from their shells and grip the substratum. In early November 2008, marked animals were recaptured (two visits to each site), photographed as described below, re-marked and returned to the field. In September 2009 the experiment was completed by returning marked individuals to the laboratory where they were photographed.

#### Juveniles collected from the field

**Figure 2:**
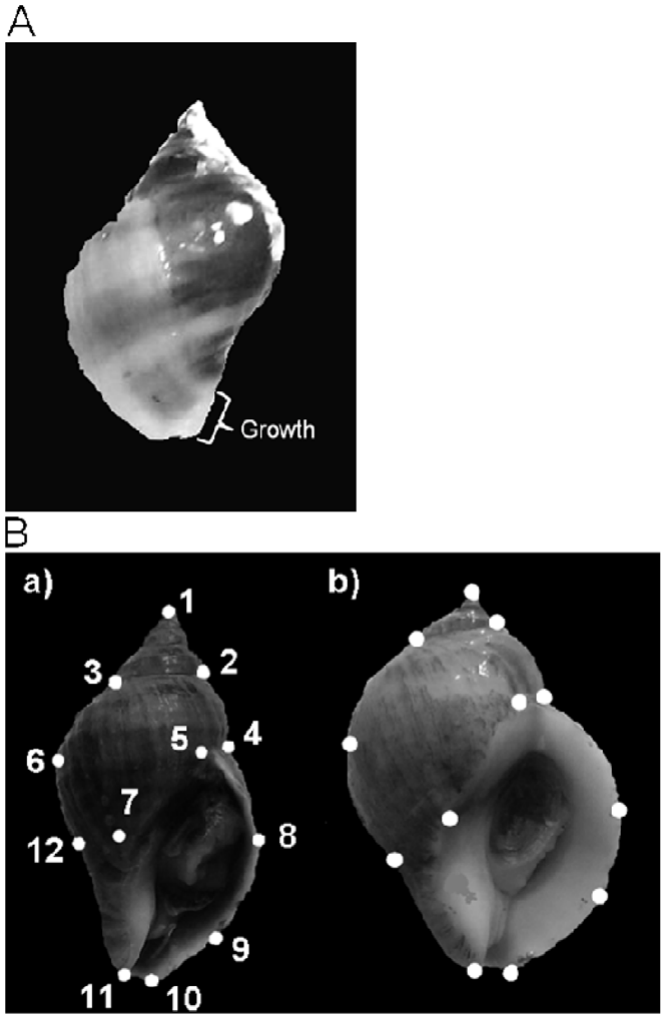
*Nucella lapillus* shell morphology. (A) Reciprocal transplant experiment: shell growth 3 months after initial release, illustrating the measured increment in shell length; (B) Morphometric analysis of *Nucella lapillus*: position of landmarks. a) shell collected from the site relatively sheltered from wave action (S Fig. 1), b) shell collected from the site exposed to strong wave action (E Fig. 1); shell length was represented by distance between landmarks1 and 11.

Initially we planned to use only laboratory-hatched young produced by adults collected from the two sites, but owing to limited yield and expected high losses in the reciprocal-transplant experiment [33], the laboratory-reared juveniles were supplemented by juveniles collected directly from the field sites. Approximately 1300 *Nucella lapillus* juveniles (≤12 mm shell length) were collected from each shore (Table 1) in early July 2008 and subjected to the same mark-recapture protocol as the laboratory-reared juveniles (above), from which they could be distinguished by colour-code. Time between collection and deployment ranged from 24 to 36 h. Unequal ratios were chosen to compensate for greater losses expected among transplants from shelter to exposure [48]. Population density within each experimental arena was conserved by relocating appropriate numbers of resident snails at a distance of about 10 m. After 3 months from initial release, callipers were used to measure the increase in shell length beyond the growth check caused by marking disturbance (Fig. 2A). Using GM software (below), shell length at the end of the experiment was measured from photographs. All snails were fixed in ethanol for subsequent use.

### Common garden experiments

Experiments were run for F_1_ and F_2_ generations (see below). Each experiment incorporated a duplicated common garden lacking the effects of wave exposure typical of the exposed field site and of crab predation typical of the sheltered field site. Quantitative comparison of traits shown by successive generations would potentially distinguish plastic, maternal and genetic components of variation.

Adults from the exposed and the sheltered site were collected in February 2008 and allowed to spawn separately in aquaria as for the reciprocal transplant experiment (above). Having grown large enough for marking (≤12 mm shell length), hatched juveniles (F_1_ generation) were apportioned between two tanks: 25 of sheltered site ancestry and 90 of exposed site ancestry per tank (fewer juveniles of sheltered site ancestry were obtained from the brood stock, causing imbalance in numbers). Seawater was supplied via two constant-head cisterns at a rate of 3 ml s^−1^ with permanent aeration supplied from air-diffusion stones. Ambient temperature fluctuated seasonally between 8–16°C. To avoid position effects, tanks were transposed each month. Subjects were photographed, as below, after 6 months and again after 12 months from the beginning of the experiment. Since eggs were laid in both tanks, opportunity was taken to continue the experiment through the F_2_ generation, retrospectively assigning parentage by genetic analysis (below).

### Morphometry

Photographic images were obtained in standard orientation (Fig. 2B), using graph paper as background for accurate scaling. Images were analysed using geometric morphometrics (GM) [49]–[51]. Twelve landmarks (Fig. 2B), nine of which had previously been employed by Guerra-Varela et al. [35], were positioned on each photographic image and analyzed using TPS [52], [53] to generate relative warps (RWs). Scores on the first three RWs were compared among treatments by MANOVA (SPSS 12.0). Deformation grids generated by TPS were used to visualize shape variation represented by the RWs. MODICOS [54] was used to obtain absolute measurements of shell length from landmarks 1 and 11 (Fig. 2B). Growth rate was compared among treatments by ANOVA. Bonferroni correction was used for multiple paired comparisons.

### Population genetics

We assessed chromosome numbers and analysed key mitochondrial and nuclear markers in each population to confirm the absence of karyotype polymorphism [44], possibly associated with phylogenetic differences that might otherwise confound adaptive phenotypic variation [34], [55].

#### Karyotype

Five juveniles from the exposed site and five from the sheltered site were karyotyped using standard protocols [46], [56]. Briefly, tissues were chopped and treated with two combined colchicine and 0.075 M KCl hypotonic treatments: 0.08% colchicine in 50% sea water for 45 min plus KCl for 30 min followed by colchicine 0.04% in 25% sea water for 45 min plus 60 min in KCl and finally fixed in Carnoy's solution (ethanol∶ acetic acid, 3∶1) at 4°C. The fixed tissues were transferred to a drop of 60% acetic acid on a slide at 40°C, where the cells were dispersed and allowed to dry before staining for 15 min in fresh, 10% Giemsa (VWR) and finally rinsing in tap water. Five to ten slides were prepared from each juvenile. Slides were examined using a Nikon microscope eclipse 50i at 1000× magnification and the clearest chromosome sets photographed for karyotyping.

#### Mitochondrial and nuclear gene amplification

Chromosome counting was complemented by comparison between populations of mitochondrial (16S) and nuclear (mMDH) genes, which vary in association with karyotypic and phenotypic polymorphism, in turn correlated with environmental variables such as wave exposure [34], [44], [57]. Six individual RNA samples from each population were analysed using the mitochondrial gene 16S and the nuclear gene mitochondrial malate dehydrogenase (mMDH). mMDH loci was amplified as described in [55]. Briefly, total RNA was extracted and DNase treated from muscle tissue using an RNeasy kit (Qiagen) followed by cDNA synthesis using the first strand cDNA synthesis kit (Fermentas). cDNA template was amplified following Kirby protocol; firstly with mMDHP1 and mMDHP2 primers and then re-amplified with the primer pair mMDHP3 and mMDHP4 in order to get a 91 bp fragment. This gene fragment was amplified once it exhibits similar differentiation levels as the complete gene amplification [57]. The mitochondrial 16S gene was amplified using the primers 16SNucFW (5′-TCTGACCTGCCCAGTGAAAT-3′) and 16SNucRV (5′-CTCAGTCGGCCCAACTAAAA-3′) (I. Colson, personal communication). PCR amplifications were carried out in 25 µl reactions containing 1 µl of cDNA, 0.3 pmol of each primer, 1× PCR Buffer, 1.5 mM MgCl_2_, 0.2 mM of each dNTP (Promega) and 0.5 U Taq DNA polymerase (Promega) on an Biorad DNA engineTetrad2 Thermal cycler. An initial denaturation step of 5 min at 95°C was followed by 35 cycles at 95°C for 1 min, 53°C for 1 min, and 72°C for 1 min followed by a final extension at 72°C for 10 min. PCR results for both genes were sequenced using the Macrogen™ (www.macrogen.com) sequencing facility and subsequently aligned and compared using the software Bioedit [58].

#### Assessment of gene flow

Microsatellites were used to assess gene flow between the two populations, separated by approximately 45 km or 90 km of coastline depending on dispersal route. Adult *N. lapillus* from Cable Bay (N = 96) and Llanfairfechan (N = 96) (Fig. 1) were collected in September 2008 and fixed in absolute ethanol. DNA was extracted from foot tissue using the CTAB (Hexadecyltrimethylammonium Bromide) DNA Extraction protocol as described in Colson and Hughes [59]. Each individual was genotyped at 9 microsatellite loci [60]. Microsatellites were amplified with the Qiagen Multiplex PCR kit following the manufacturer's instructions using two different primer mixes: Nlw2, Nlw3, Nlw8, and Nlw14 in the first mix and Nlw11, Nlw17, Nlw21, Nlw25 and Nlw27 in the second mix. With slightly differences to the PCR reaction and program, the fluorescent M13 tail single-reaction nested PCR method [61] was used to amplify the loci. An initial denaturation step of 15 minutes at 95°C was followed by 13 cycles at 94°C for 30 s, 55°C for 90 s, and 72°C for 60 s. In order to attach the dye tails to the PCR product, an extra 31 cycles at 94°C of 30 s, 50°C for 90 s and 72°C for 60 s were performed and followed by a final extension at 60°C for 30 minutes). Extension products were resolved on an ABI 3130xl (Applied Biosystems) and alleles were sized to an internal size standard (GeneScan-500 LIZ; Applied Biosystems) using the GeneMapper software version 4.0 (Applied Biosystems). Raw data were screened using GenAlEx version 6.2 [62] and Micro-checker [63] to avoid scoring errors. Tests for deviations from Hardy-Weinberg proportions, heterozygote deficiencies, genotypic linkage equilibrium and genetic heterogeneity among populations were estimated using the exact test of GENEPOP version 3.4 [64]. Allelic frequencies, mean number of alleles per locus, observed (H_0_) and expected heterozygosity (H_E_) under Hardy-Weinberg assumptions, estimates of *F_ST_*, *F_IS_*, and their significance per population over all loci were calculated according to Weir and Cockerham [65] using FSTAT version 2.9.3.2 [66].

### Parental analysis

Potential parents of known gender (F_1_, n = 112) and offspring (F_2_, n = 112) were genotyped as described above, using seven microsatellite markers (Nlw2, Nlw3, Nlw8, Nlw11, Nlw21, Nlw25 and Nlw27). Parental analysis was performed using CERVUS [67].

### Permissions

No specific permits were required for the described field studies, which took place on shores with public right-of-way and did not involve endangered or protected species.

## Results

### Reciprocal transplant experiment

#### Recapture rate

**Figure 3:**
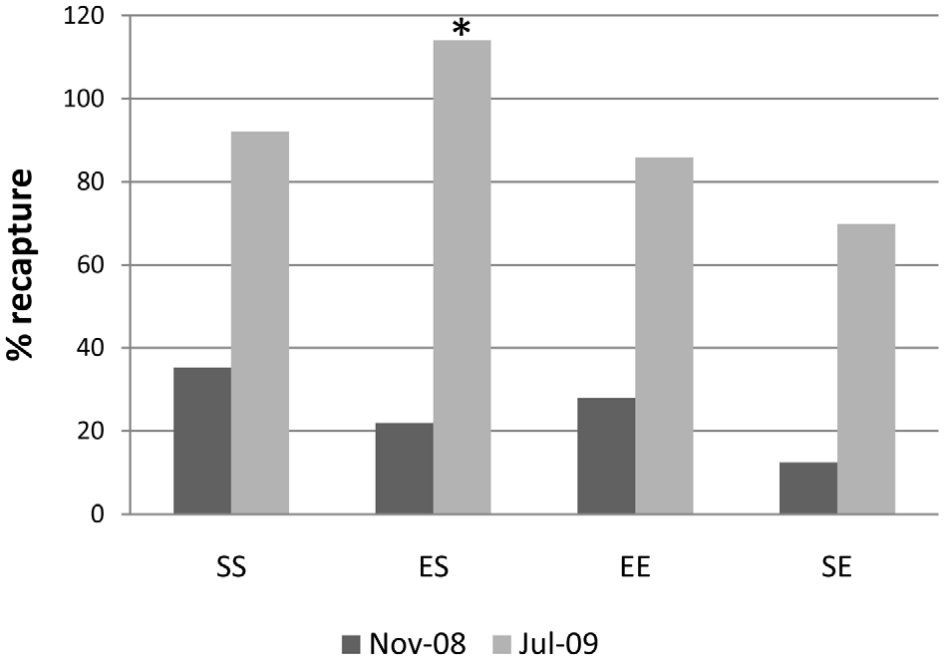
Recapture rates. Nov-08 = percentage of snails released at the beginning of the experiment (August 2008) and recaptured in November 2008; Jul-09 = percentage snails re-released in November 2008 and recaptured at the end of the experiment in July 2009. E: exposed; S:sheltered; SS (nNov = 214; nJul = 197); ES (nNov = 178; nJul = 203); EE (nNov = 226; nJul = 194); SE (nNov = 133; nJul = 93). * recapture rate>100% reflects the recapture of snails released in August 2008 but missed in November 2008.

Percentages of snails released in July 2008 and recaptured in July 2009 (Fig. S1) were ranked as follows: snails reared as juveniles in the laboratory, exposed returned to exposed site (EE)>exposed transplanted to sheltered site (ES)>sheltered returned to sheltered site (SS)>sheltered transposed to exposed site (SE); snails collected as juveniles from the field, SS>ES>EE>SE. The difference in ranking between laboratory-hatched and field-collected snails reflects the greater initial mortality of laboratory-hatched, sheltered-site snails, probably caused inadvertently during marking. Pooled data for laboratory-reared and field-collected juveniles (Fig. 3) show that relatively low percentages of snails released in July 2008 were recaptured in November 2008, but much higher percentages of snails re-released in November 2008 were recaptured in July 2009. Recapture rate for SE was lower than for any other treatment over both the first four and second eight months of the experiment. Recapture rate for treatment ES was comparable to EE and less than SS during the first four months, but was greater than SS during the second eight months of the experiment.

#### Shell growth

**Figure 4:**
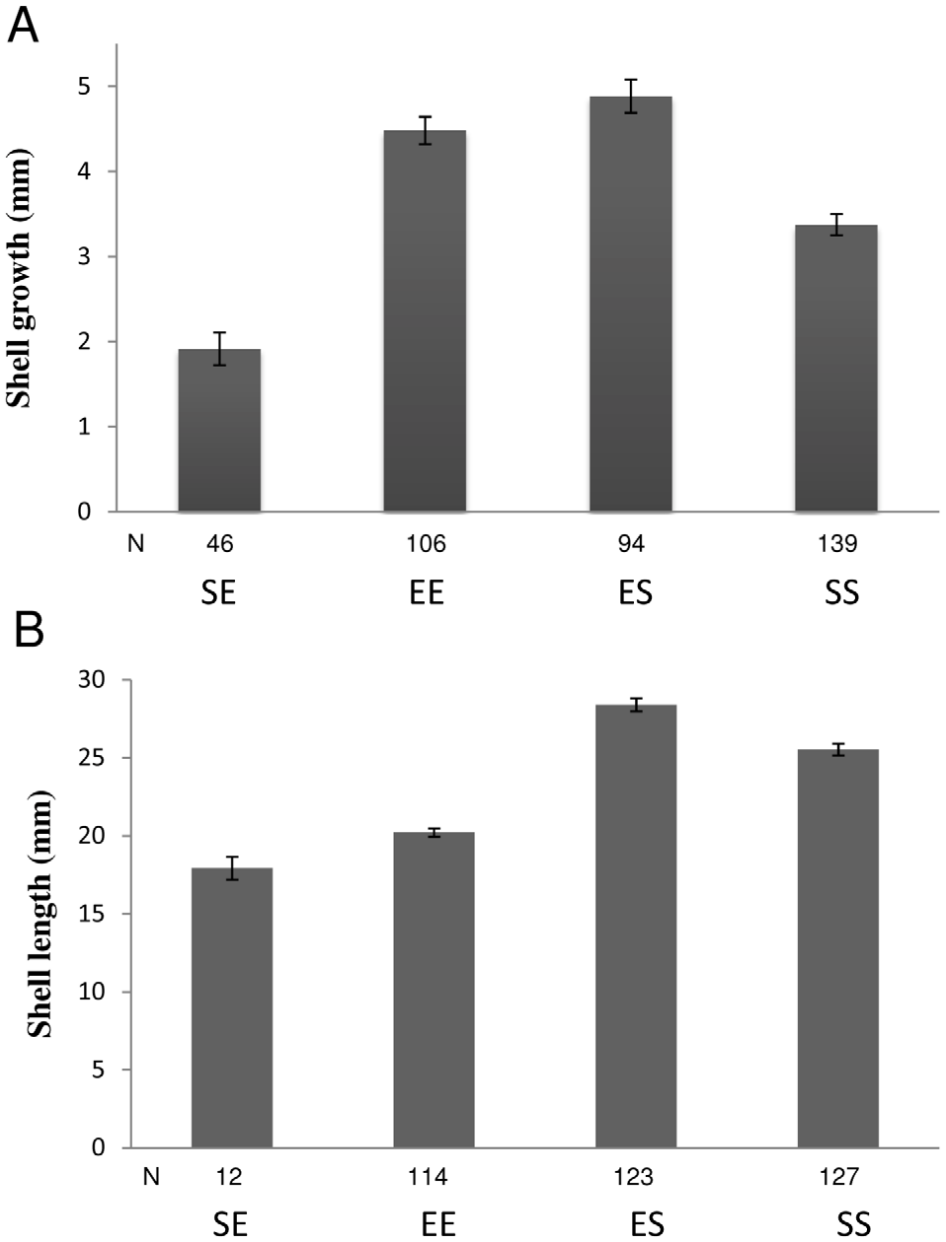
Shell growth. (A) Growth after 3 months in the field. (B) Shell length after 12 months in the field. SS = sheltered-site snails (controls) replanted at the sheltered site, SE = sheltered-site snails transplanted to the expose site, EE = exposed-site snails (controls) replanted at the sheltered site, ES = exposed-site snails transplanted to the sheltered site. Data are means with standard errors. Sample sizes are given below the bars.

Initial shell length did not differ significantly between snails of exposed-site and sheltered-site ancestry (mean sheltered = 10.0 mm, S.E. = 0.9 mm; mean exposed = 9.3 mm, S.E. = 0.5 mm; t = 1.014; P = 0.321). Shell growth at 3 months after initial release was ranked ES>EE>SS>SE (Fig. 4A, ANOVA, all paired comparisons P<0.001). By the end of the experiment, initial growth checks had become obscured, preventing measurement of incremental growth. After 12 months in the field, however, shell length was ranked ES>SS>EE>SE (Fig. 4B, ANOVA, all paired comparisons P<0.001).

#### Shell morphology

**Figure 5:**
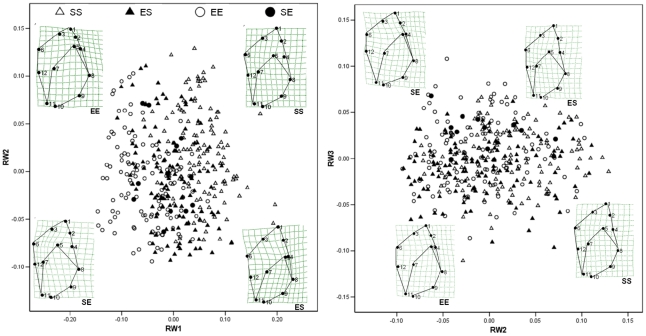
Phenotypic plasticity. GM analysis of shells grown for 12 months in the field. Data are RW scores standardized for centroid size. Deformation grids correspond to individuals subjectively chosen to represent the central tendency in each treatment. Treatment-labels are as in Fig. 4.

RW1, RW2 and RW3 accounted for 32%, 19% and 10% of variance respectively. Since RWs 1–3 were correlated with centroid size (Pearson r, P<0.05), scores were standardized as residuals from linear regression. All paired comparisons (Table 2) were statistically significant on at least one RW, although the power of comparisons involving SE was reduced by small sample size. Deformation grids show that RWs 1–3 represented variation in globularity of the shell and relative size and shape of the aperture (Fig. S2). Exposed-site controls had more globular shells, with shorter, blunter spires and larger, wider apertures than sheltered-site controls, while reciprocal transplants were intermediate in shape (Fig. 5).

**Table 2:** Reciprocal transplant experiment: differences in shell shape among treatments.

Treatment	EE	SE	ES
SS	**<0.001**	**<0.001**	**<0.001**
	**<0.001**	0.752	**<0.001**
	0.328	**0.017**	0.133
EE		**0.027**	**<0.001**
		>0.999	>0.999
		0.182	**<0.001**
SE			>0.999
			>0.999
			**0.001**

Paired comparisons of mean standardized scores on RWs 1–3 (MANOVA: Box's M, P = 0.068; Wilk's lambda, P<0.001; Levene's test: RW1, P = 0.160; RW2, P = 0.470; RW3, P = 0.003). Treatment-symbols are as in Fig. 4. Sample sizes: SS = 128, EE = 114, SE = 12, ES = 122.

### Common garden experiment: heritable variation in shell morphology

F_1_ parents comprised 36 males and 54 females of exposed-site ancestry and 10 males and 14 females of sheltered-site ancestry. Of the F_2_ progeny, 112 snails survived for 12 mo and genotyping unequivocally assigned 85 of these to known parentage, 5 having sheltered-site ancestry, 52 exposed-site ancestry and 28 mixed ancestry. The P generation was represented by controls from the reciprocal transplant experiment.

**Figure 6:**
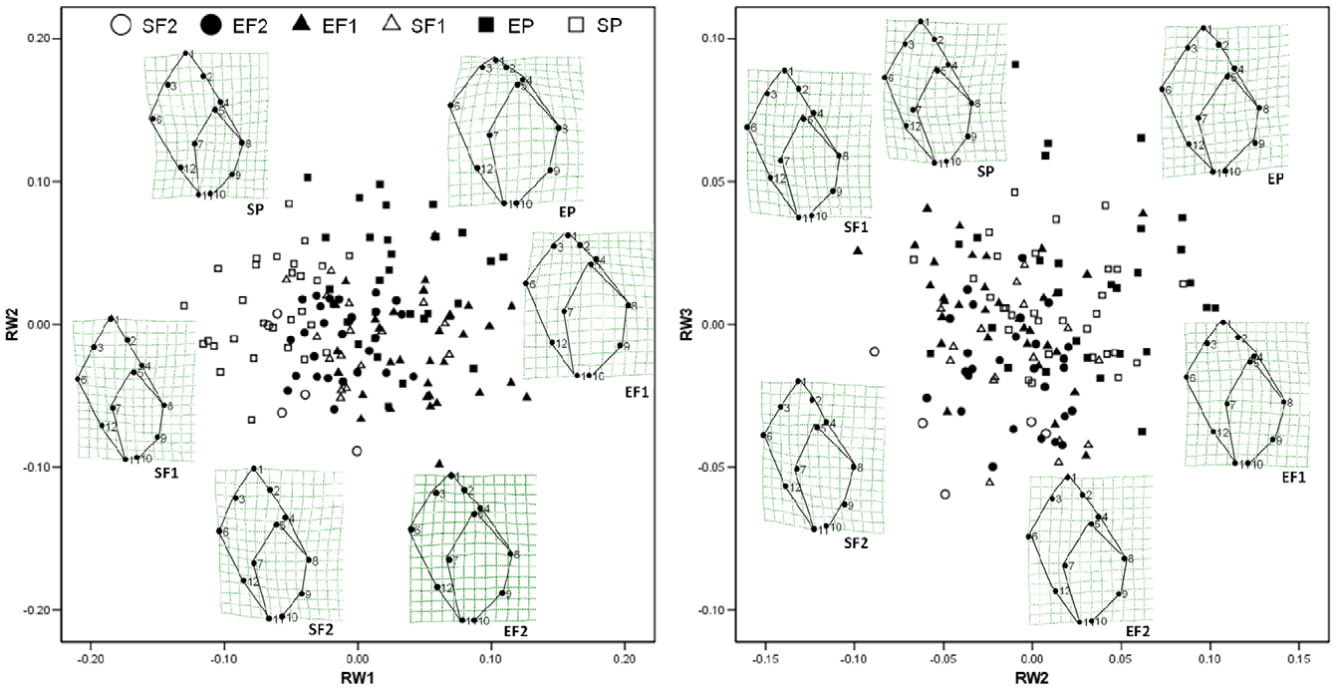
Heritable phenotypic variation. GM analysis of shells grown for 12 months in the field (P generation) or for 12 months in common garden (F_1_ and F_2_ generations). EP = exposed P generation, EF_1_ = exposed F_1_ generation, SP = sheltered P generation, SF_1_ = sheltered F_1_ generation, EF_2_ = exposed F_2_ generation, SF_2_ sheltered F_2_ generation. Data and deformation grids are as in Fig. 5.

RW1, RW2 and RW3 (Fig. 6) accounted for 35%, 16% and 15% of variance respectively. Mean scores on at least one RW differed between sheltered- and exposed site snails in the P and F_1_ generations but not in the F_2_ generation except by planned comparison (Table 3). Mean scores also differed between generations within lineages, except sheltered F_1_–F_2_ (Table 3). Deformation grids show that differences in shape between exposed- and sheltered-site lineages became less pronounced in successive generations, yet remained discernible even in the F_2_ (Fig. 6).

**Table 3:** Differences in shell shape among generations.

Treatment	EP	SF_1_	EF_1_	SF_2_	EF_2_
SP	**<0.001**	**<0.001**	**<0.001**	>0.999	**<0.001**
	0.476	0.804	**0.006**	**0.039**	0.211
	>0.999	**0.029**	>0.999	**0.001**	**0.001**
EP		0.081	0.516	**<0.001**	**<0.001**
		**0.004**	**<0.001**	**0.001**	**<0.001**
		**0.001**	>0.999	**<0.001**	**<0.001**
SF_1_			**<0.001**	0.174	>0.999
			<0.999	<0.999	>0.999
			0.063	0.942	>0.999
EF_1_				**<0.001**	**<0.001**
				>0.999	**<0.001**
				**0.003**	**0.004**
SF_2_					0.704
					>0.999
					>0.999

Post-hoc comparisons of mean standardized scores on RWs 1–3 (MANOVA: Box's M, P = 0.382; Wilk's lambda, P<0.001; Levene's test: RW1, P = 0.569; RW2, P = 0.042; RW3, P = 0.179). Treatment-symbols are as in Fig. 6. Random subsets of available snails were used for certain treatments to help balance sample sizes: SP = 30, EP = 28, SF_1_ = 16, EF_1_ = 33, SF_2_ = 5, EF_2_ = 27. Planned comparison of SF_2_ and EF_2_: RW1, P = 0.047; RW2, P = 0.094; RW3, P = 0.074.

To gain resolution within generations, GM analyses were run separately for the full F_1_ and F_2_ data sets. For the F_1_ generation, RWs 1–3 accounted for 35%, 25% and 9% of variance respectively and scores were uncorrelated with centroid size. Mean scores of sheltered-site (n = 16) and exposed-site snails (n = 51) differed on RW1 but not on RW2 or RW3, although difference on the latter was marginally non-significant (MANOVA: Box's M, P = 0.894; Wilk's lambda,P<0.001; Levene's test: RW1, P = 0.400; RW2, P = 0.032; RW3, P = 0.250. Paired comparisons: RW1, P<0.001; RW2, P = 0.194; RW3, P = 0.062). For the F2 generation, RWs 1–3 accounted for 30%, 20% and 11% of variance respectively. RW1 was correlated with centroid size and therefore corrected scores were used for subsequent analysis. Uncorrected scores were used for RW2 and RW3. Difference in mean score between sheltered-site (n = 5) and exposed-site snails (n = 27) was marginally non-significant on RW1 and RW2, and non-significant on RW3 (MANOVA: Box's M, P = 0.599; Wilk's lambda, P = 0.067; Levene's test: RW1, P = 0.231; RW2, P = 0.103; RW3, P = 0.432. Paired comparisons: RW1, P = 0.075; RW2, P = 0.085; RW3, P = 0.589).

### Karyotype and population genetics

Chromosome counts of 2n = 26–28 were obtained for both the exposed- and sheltered-site populations, which also had identical 16S sequences and possessed only one mMDH haplotype (mMDH9). There was therefore no evidence of karyotype polymorphism. All microsatellite loci were polymorphic for both populations. The number of alleles per population per locus ranged from 2 to 19, with a total number of 108 alleles in the global sample. The expected heterozygosity (H_E_) per locus ranged from 0.332 to 0.892 and the observed heterozygosity (H_O_) from 0.313 to 0.917 (Table 4). A test for concordance with HWE revealed deviations from HWE in locus Nlw2 and Nlw14 (Table 4). No evidence of linkage disequilibrium was observed between loci. Global F_IS_ was −0.0129 suggesting an excess of heterozygotes in the sampling areas. F_ST_ values per locus ranged from −0.0031 and 0.1491 and the global F_ST_ was 0.038 (P = 0.001), revealing significant structuring between the two sampling sites (Table 4).

**Table 4:** Microsatellite analysis: genetic variability measures by locus for each population.

	Exposed				Sheltered				ALL	
	Na	Ho	He	HWE	Na	Ho	He	HWE	F_IS_	F_ST_
**Nlw2**	9	0.906	0.827	0.000	10	0.917	0.821	0.000	−0.101	0.021
**Nlw3**	9	0.760	0.774	0.595	7	0.583	0.515	0.059	−0.037	0.149
**Nlw8**	17	0.844	0.841	0.880	19	0.906	0.892	0.225	−0.005	0.007
**Nlw11**	12	0.792	0.790	0.640	13	0.792	0.830	0.269	0.028	0.077
**Nlw14**	13	0.719	0.850	0.000	14	0.917	0.848	0.003	0.042	0.024
**Nlw17**	12	0.802	0.867	0.004	15	0.885	0.878	0.003	0.038	0.008
**Nlw21**	2	0.344	0.359	0.776	3	0.313	0.332	0.622	0.055	0.008
**Nlw25**	4	0.448	0.378	0.324	4	0.448	0.476	0.673	−0.044	0.021
**Nlw27**	7	0.719	0.628	0.306	8	0.594	0.556	0.709	−0.103	0.006
**All**									−0.0129	0.0376

Na: number of alleles found per locus; H_E_: expected heterozygosity; H_O_: observed heterozygosity; F_IS_: standardised genetic variance within populations at each locus; F_ST_: standardized genetic variance among populations at each locus; HWE: Hardy-Weinberg P values.

## Discussion

### Recapture rate

In the reciprocal transplant experiment, losses in all treatments occurred mostly within four months of initial release. Although care was taken to minimise physiological stress, it is likely that the procedures of marking and release amplified intrinsic vulnerability associated with small size. Markedly fewer losses occurred once individuals had become established and grown larger. Recapture rate was clearly lowest among sheltered-site snails transplanted to the exposed site and probably attributable to periods of intense wave action that frequently occurred during the experiment. In striking contrast to the above, recapture rate over the second eight months of the experiment of snails transplanted from the exposed site to the sheltered was not only greater than that of controls at the exposed site but even greater than controls at the sheltered site. This unexpected result may reflect resistance to crab predation accruing to exposed-site snails through rapid growth to a size-refuge, discussed below, and through greater shell thickening (unpublished data).

### Growth rate

*Nucella lapillus* is reported to have higher size-specific somatic growth rate but similar size-specific shell-growth rate at exposed sites than at sheltered [24], [68]. In contrast to specific growth rate, cumulative growth of body and shell are reported to be greater at sheltered sites than exposed [68], [69]. Moreover, snails we transplanted from the exposed site grew larger shells than sheltered-site controls (somatic growth was not measured), suggesting a heritable component promoting higher specific shell-growth rate in snails of exposed-site provenance. We have no evidence of heritable differences in prey-handling ability between populations [70] and conclude that the relatively small adult size attained by exposed-site snails probably resulted from reduced foraging opportunity imposed by wave action. In corroboration, snails transplanted from the sheltered to the exposed site grew slower and to a smaller size than in any other treatment, commensurable with the influences of shorter cumulative foraging time and lower specific growth rate. Similar variation in growth rate has also been reported for *Littorina obtusata*, in which snails from exposed sites grew faster than those from sheltered under laboratory conditions of low flow velocity [71] and for *L. saxatilis* where snails transplanted from high to low shore grew faster than low-shore residents [72]. The above trends in growth rate perhaps may be explained respectively in terms of physiological compensation for constrained foraging time imposed by wave action [73] or rapid attainment of a size-refuge from crab predation [74]. Positive association between drag, risk of dislodgement and shell-size has also been invoked to explain in evolutionary terms the smaller maximum size of *Nucella* spp. observed at exposed sites (e.g. [75]–[77]). In the exposed-site population studied here, however, high growth potential revealed by transplantation to the sheltered site indicates that smaller maximum size at the exposed site is the result of environmental constraint rather than natural selection. Rapid growth to a size-refuge from crab predation [28], [71], [74] may explain the high survivorship of snails transplanted from the exposed to the sheltered site. The size-refuge hypothesis was invoked by Johannesson et al. [74] to explain the evolution of intrinsically faster growth of *Littorina saxatilis* from crab-infested habitat compared with those from crab-free habitat. In our study, however, *N. lapillus* showed the opposite trend and we propose that intrinsically faster growth in snails from the crab-free, exposed site is selectively advantageous by maximizing cumulative growth over the limited periods when reduced wave action permits foraging [73]. Higher specific growth rate thus may have been of secondary advantaged to snails transplanted from the exposed to the sheltered site.

### Plastic and heritable components of variation in shell shape

Our data augment those of Etter [33] by demonstrating partial convergence of phenotype, in our case shell-shape and in his pedal area, toward that of exposed-site residents. In contrast to Etter [33], however, our data also clearly demonstrate phenotypic convergence of transplanted exposed-site snails toward sheltered-site residents. The apparent discrepancy may reflect choice of phenotypic traits and methodology. Owing to the influence of multiple selection forces discussed above and the probability of some independent variation among traits, correlation between pedal area and shell shape is likely to be partial. Moreover, supplementary data (Data S1) show that differentiation of shell-shape between snails of exposed and sheltered ancestry may already be discernible at shell lengths of 5–6 mm corresponding to 4–5months of age (Fig. S3, S4). Smaller initial size (≤12 mm v. ≥14 mm) and longer duration (12 mo v. 5 mo) may have lessened any effect of prior ontogeny and allowed greater scope for morphological divergence in our experiment. Our results therefore complement rather than contradict those of Etter [33].

Differentiation of shell-shape between exposed- and sheltered-site lineages observed in the field (P generation) persisted into the F_1_ generation raised in the laboratory. With the caveat that power of analysis was compromised by small sample-size for sheltered-site snails, we cautiously conclude that differentiation also persisted into the F_2_ generation. Differentiation, however, became progressively weaker in successive generations. Reduced lineage-differentiation between the P and F_1_ generations may have been attributable to plastic convergence, although lineages adapted to different suites of selection forces at exposed and sheltered sites may not necessarily perceive a common laboratory environment in the same way. Selection seems an unlikely cause of convergence as both lineages survived well and reproduced freely when brought from the field into the laboratory. Reduced lineage-differentiation between the F_1_ and F_2_ generations was probably due to diminished maternal effects [78]. Residual differentiation of lineages within the F_2_ generation, however, indicates that the characteristic difference in shell-shape between our field populations is controlled genetically as well as by maternal effects and plasticity. Moreover, our data suggest that genetic control of phenotypic differentiation in *N. lapillus* can occur in the absence of karyotype polymorphism, when it is likely to reflect local adaptation rather than phylogenetic constraint. Magnitude of divergence at microsatellite loci (Table 4) coincides with predicted levels for marine taxa lacking pelagic larvae [79], [80] and shows that the two populations were genetically semi-isolated. Because we used neutral markers, however, we could not distinguish between the possible influences of drift or selection on differentiation of the two populations. Nevertheless, the occurrence of heritable phenotypic variation among all populations studied (above results; Data S1, Fig. S3, S5) suggests that local selection may be involved, especially since it is well established that depending on demographic factors, local adaptation is often promoted in genetically discrete populations [81]. Rank correlation between heritable phenotype and wave exposure at ancestral sites that include extremes and intermediate levels of exposure (Fig. S5) is probably superficial, however, in the sense that although selectively important physical variables associated with exposure to wave action may vary monotonically among shores, other selection forces including risk of crab predation may be typified by more complex spatial variation.

We have shown that in two populations, at least, adaptive phenotypic variation is controlled by developmental plasticity, maternal effects and probably by genotype. Despite lack of replication of our observations across other shores, their concordance both with theoretical predictions, and with trends from other published studies [2], [16], [33], [35], supports strongly the generality of findings. Matching of phenotype to environment by selection would be the most effective promoter of fitness if the environment experienced by parents and offspring were highly predictable. *N. lapillus* aggregate in protective microhabitats in order to mate and spawn and although lacking specific homing behaviour they tend to use a restricted number of spawning sites distributed within a radius of up to about10 m that also encompasses their foraging range ([36], personal observation). Even on such a local scale, however, cliffs or reefs with large crevices and blocks presenting microhabitats exposed to and sheltered from major wave impact might provide a template for fine-scale population genetic structuring, as recorded by other studies of *N. lapillus* [13], [16], [35] and as extensively demonstrated for populations of *Littorina saxatilis* [15], [82]–[84]. Movement into new local habitat may occur through diffusion during successive foraging cycles [73], [85] and by dislodgement and transportation by waves. Yet despite typically localised movement, *N. lapillus* is also capable of dispersal over several kilometres or more, probably by early juveniles drifting while attached to buoyant mucous threads or debris [59], [86]. Synergistic genetic, maternal and plastic control of phenotype will maximise fitness when environmental circumstances become uncertain due to vagaries of local or long-distance dispersal.

## Supporting Information

Figure S1 **Recapture rates.** Nov-08 = percentage of snails released at the beginning of the experiment (August 2008) and recaptured in November 2008; Jul-09: percentage of snails released at the beginning of the experiment and recaptured at the end of the experiment in July 2009; Jul-09b = percentage snails re-released in November 2008 and recaptured at the end of the experiment. E: exposed; S:sheltered; f: juveniles collected from the field; l: laboratory-hatched juveniles; fSS (nNov = 203; nJul = 185); fES (nNov = 153; nJul = 160); fEE (nNov = 169; nJul = 142); fSE (nNov = 109; nJul = 71); lSS (nNov = 11; nJul = 12); lES (nNov = 25; nJul = 43); lEE (nNov = 57; nJul = 52); lSE (nNov = 24; nJul = 22).
(TIF)

Figure S2 **Reciprocal transplant experiment: extreme deformation grids – Rw1/Rw2 (top), Rw2/Rw3 (bottom).**
(TIF)

Figure S3 **Location of study sites along the North Wales coastline.** CB = Cable Bay (53°12.410′N, 04°30.290′W), Thomas Exposure Index (TEI) = 14; CAE = Caethle (53°11.212′N, 04°30.249′W), TEI = 15; FB = Friars Bay (53°16.107′N, 04°05.113′W), TEI = 3; LL = Llanfairfechan (53°15.769′N, 03°55.142′W), TEI = 2; MB = Menai Bridge (53°13.272′N, 04°09.861′W), TEI = 0; RP = Ravens Point (53°16.161′N, 04°37.548′W), TEI = 14; RWB = Redwharf Bay (53°18.594′N, 04°08.495′W), TEI = 8.
(TIF)

Figure S4 **Experiment 1: ontogenetic changes in shell morphology.** Mean aperture external width adjusted to shell length (ANCOVA). For each size class, the first group represents snails reared in the laboratory and the second group snails collected from the ancestral field-population. Sample size (N) and covariate value (CV) for adjusting mean aperture width were as follows. Group 1: N exposed 1 = 10, N exposed 2 = 15, N sheltered 1 = 11, N sheltered 2 = 12; CV = 2.264 mm. Group 2: N exposed 1 = 11, N exposed 2 = 12, N sheltered 1 = 15, N sheltered 2 = 15; CV = 2.726 mm. Group 3: N exposed 1 = 13, N exposed 2 = 14, N sheltered 1 = 12, N sheltered 2 = 6; CV = 7.915 mm. Group 4: N exposed 1 = 15, N exposed 2 = 15, N sheltered 1 = 15, N sheltered 2 = 15; CV = 7.670 mm. Group 5: N exposed 1 = 17, N exposed 2 = 17, N sheltered 1 = 15, N sheltered 2 = 18; CV = 12.331 mm. Group 6: N exposed 1 = 15, N exposed 2 = 15, N sheltered 1 = 15, N sheltered 2 = 15; CV = 12.753 mm. Group 7: N exposed 1 = 35, N exposed 2 = 30, N sheltered 1 = 28, N sheltered 2 = 22; CV = 18.066 mm. Group 8: N exposed 1 = 39, N exposed 2 = 30, N sheltered 1 = 30, N sheltered 2 = 30; CV = 16.872 mm.
(TIF)

Figure S5 **Comparison of relative aperture width of adult laboratory-reared and field-collected** ***N. lapillus*** **from shores differing in exposure to wave action.** Adjusted means (see text) are shown with standard errors. Shores are ranked in increasing order of wave exposure (see Fig. S3): Menai Bridge (Thomas exposure index (TEI) = 0; Llanfairfechan, TEI = 1; Friars Bay, TEI = 4; Redwharf Bay, TEI = 8; Ravens Point, TEI = 13; Cable Bay, TEI = 14; Caethle, TEI = 15.
(TIF)

Data S1 **Supplementary data.** Experiment 1: ontogenetic changes in shell morphology; Experiment 2: wave-exposure gradient.
(DOC)
