# Supplementary figures and images for: Plastic and Heritable Components of Phenotypic Variation in Nucella lapillus: An Assessment Using Reciprocal Transplant and Common Garden Experiments
Source: PLoS One. 2012 Jan 27;7(1):e30289. doi: 10.1371/journal.pone.0030289 (PMC3267715; doi:10.1371/journal.pone.0030289)

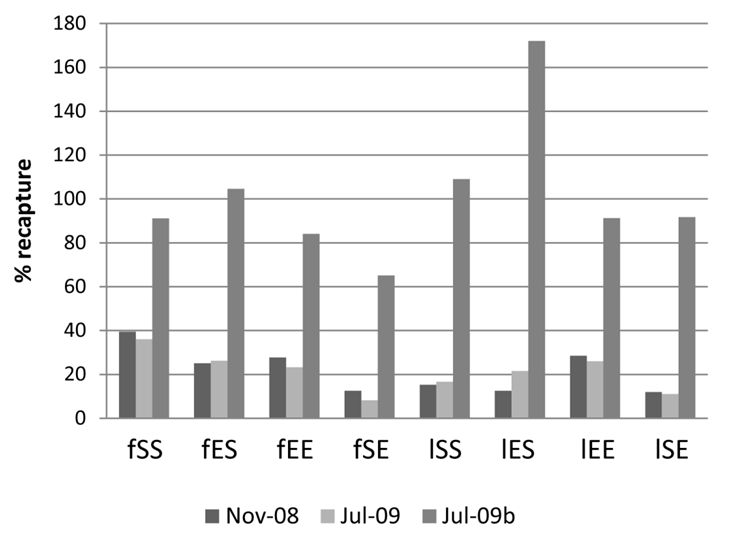

Supplement: Figure S1 — Recapture rates. Nov-08 = percentage of snails released at the beginning of the experiment (August 2008) and recaptured in November 2008; Jul-09: percentage of snails released at the beginning of the experiment and recaptured at the end of the experiment in July 2009; Jul-09b = percentage snails re-released in November 2008 and recaptured at the end of the experiment. E: exposed; S:sheltered; f: juveniles collected from the field; l: laboratory-hatched juveniles; fSS (nNov = 203; nJul = 185); fES (nNov = 153; nJul = 160); fEE (nNov = 169; nJul = 142); fSE (nNov = 109; nJul = 71); lSS (nNov = 11; nJul = 12); lES (nNov = 25; nJul = 43); lEE (nNov = 57; nJul = 52); lSE (nNov = 24; nJul = 22). (TIF) [file pone.0030289.s001.tif]

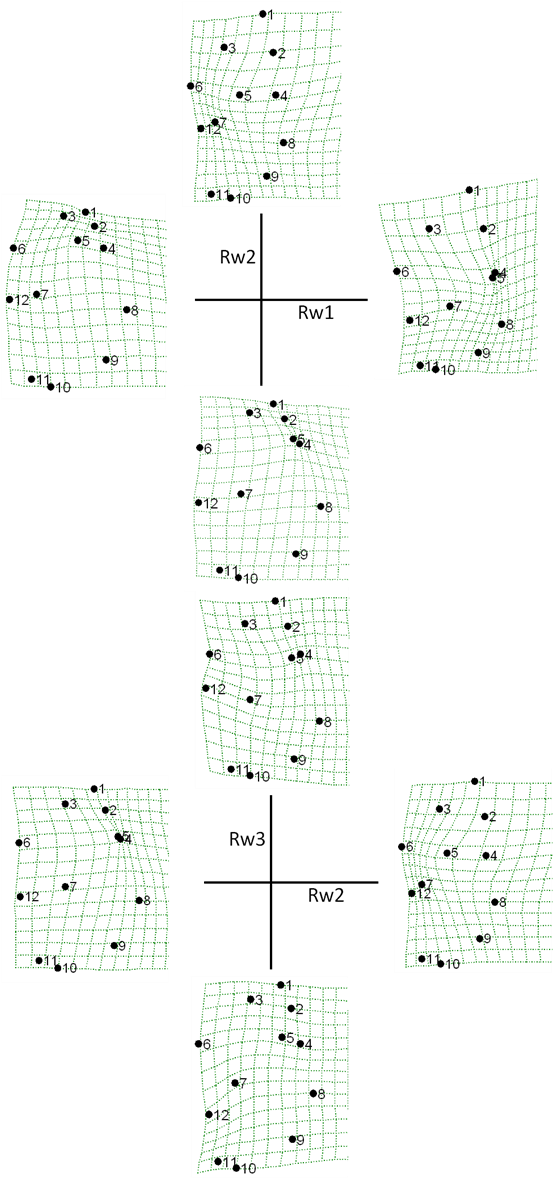

Supplement: Figure S2 — Reciprocal transplant experiment: extreme deformation grids – Rw1/Rw2 (top), Rw2/Rw3 (bottom). (TIF) [file pone.0030289.s002.tif]

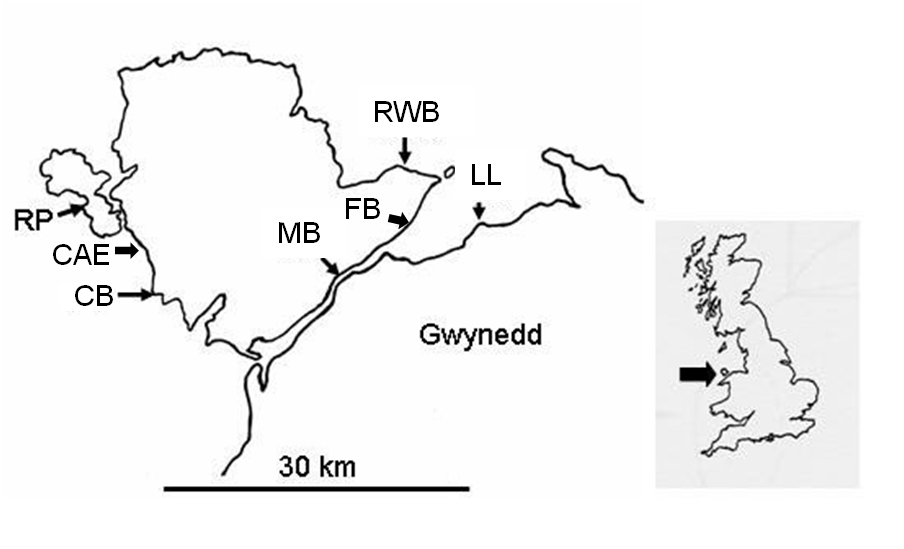

Supplement: Figure S3 — Location of study sites along the North Wales coastline. CB = Cable Bay (53°12.410′N, 04°30.290′W), Thomas Exposure Index (TEI) = 14; CAE = Caethle (53°11.212′N, 04°30.249′W), TEI = 15; FB = Friars Bay (53°16.107′N, 04°05.113′W), TEI = 3; LL = Llanfairfechan (53°15.769′N, 03°55.142′W), TEI = 2; MB = Menai Bridge (53°13.272′N, 04°09.861′W), TEI = 0; RP = Ravens Point (53°16.161′N, 04°37.548′W), TEI = 14; RWB = Redwharf Bay (53°18.594′N, 04°08.495′W), TEI = 8. (TIF) [file pone.0030289.s003.tif]

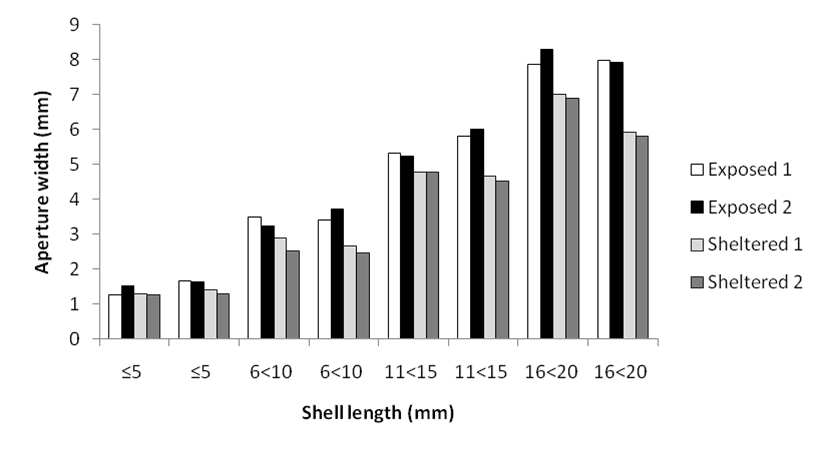

Supplement: Figure S4 — Experiment 1: ontogenetic changes in shell morphology. Mean aperture external width adjusted to shell length (ANCOVA). For each size class, the first group represents snails reared in the laboratory and the second group snails collected from the ancestral field-population. Sample size (N) and covariate value (CV) for adjusting mean aperture width were as follows. Group 1: N exposed 1 = 10, N exposed 2 = 15, N sheltered 1 = 11, N sheltered 2 = 12; CV = 2.264 mm. Group 2: N exposed 1 = 11, N exposed 2 = 12, N sheltered 1 = 15, N sheltered 2 = 15; CV = 2.726 mm. Group 3: N exposed 1 = 13, N exposed 2 = 14, N sheltered 1 = 12, N sheltered 2 = 6; CV = 7.915 mm. Group 4: N exposed 1 = 15, N exposed 2 = 15, N sheltered 1 = 15, N sheltered 2 = 15; CV = 7.670 mm. Group 5: N exposed 1 = 17, N exposed 2 = 17, N sheltered 1 = 15, N sheltered 2 = 18; CV = 12.331 mm. Group 6: N exposed 1 = 15, N exposed 2 = 15, N sheltered 1 = 15, N sheltered 2 = 15; CV = 12.753 mm. Group 7: N exposed 1 = 35, N exposed 2 = 30, N sheltered 1 = 28, N sheltered 2 = 22; CV = 18.066 mm. Group 8: N exposed 1 = 39, N exposed 2 = 30, N sheltered 1 = 30, N sheltered 2 = 30; CV = 16.872 mm. (TIF) [file pone.0030289.s004.tif]

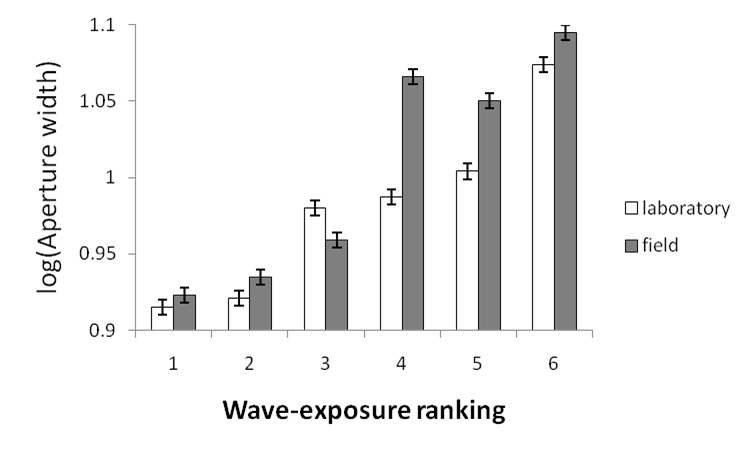

Supplement: Figure S5 — Comparison of relative aperture width of adult laboratory-reared and field-collected N. lapillus from shores differing in exposure to wave action. Adjusted means (see text) are shown with standard errors. Shores are ranked in increasing order of wave exposure (see Fig. S3): Menai Bridge (Thomas exposure index (TEI) = 0; Llanfairfechan, TEI = 1; Friars Bay, TEI = 4; Redwharf Bay, TEI = 8; Ravens Point, TEI = 13; Cable Bay, TEI = 14; Caethle, TEI = 15. (TIF) [file pone.0030289.s005.tif]
