# Supplementary material for: Plastic and Heritable Components of Phenotypic Variation in Nucella lapillus: An Assessment Using Reciprocal Transplant and Common Garden Experiments
Source: PLoS One. 2012 Jan 27;7(1):e30289. doi: 10.1371/journal.pone.0030289 (PMC3267715; doi:10.1371/journal.pone.0030289)
Supplement: Data S1 — Supplementary data. Experiment 1: ontogenetic changes in shell morphology; Experiment 2: wave-exposure gradient. (DOC) [file pone.0030289.s006.doc]

**Supplementary data: Data S1**

**Experiment 1: ontogenetic changes in shell morphology.**

*N. lapillus* was sampled from four shores in North Wales (Fig. S3). Two shores (Cable Bay and Ravens Point) represented habitat exposed to strong wave action generated by prevailing south-westerly winds blowing from the English Channel and across the Irish Sea, while the other two (Menai Bridge, Llanfairfechan) represented more sheltered habitat in the lee of the prevailing winds. Geographical coordinates and an index combining mean annual wind energy and fetch to quantify wave exposure [87] are given in the legend to Fig. S3. Aggregations of spawning adults were located at low-spring-tide level in February 1997. Samples of 50 adults and about 100 egg capsules were collected per shore. Care was taken to collect egg capsules from several different positions within a clump, in order to increase the likelihood of obtaining progeny from a number of parents. Adults were fixed in alcohol and egg capsules were transferred to laboratory aquaria measuring 60 x 40 x 30cm and dedicated one per population to avoid any possibility of mixing, as might occur using meshed cages within aquaria. Egg capsules were inaccessible at Ravens Point and so 100 adults were collected and maintained in an aquarium, where they spawned. To ensure absence of crab effluent, aquaria were provisioned with seawater obtained from Cable Bay North where crabs are absent intertidally. The seawater was renewed bimonthly, gently aerated and allowed to follow ambient temperature within a seasonal range of about 10-18°C. Evaporation was minimized by placing clear plastic sheeting over the aquaria. Small mussels, below 10mm in shell length, were provided as prey and renewed as needed to maintain unlimited availability to hatched snails. Aquaria were reshuffled at each water change to avoid position effects. Measurements were taken of shell length (landmarks 1-11 in Fig. 2B) and aperture external width (landmarks 7-8). Samples of 15 snails per population were fixed in alcohol and measured under a dissection microscope when they had reached a shell height of about 4mm, approximately 3 months after hatching. Independent, successive samples of 30 snails were fixed and measured with callipers to 0.05mm at 8, 12, 18 and 23 months after hatching. Ontogenetic changes in shell shape of native snails were quantified using samples of 50 individuals in successive size classes 1-5, 6-10, 11-15 and 16-20mm shell height collected in March 1998. Differentiation of shell morphology among snails from exposed and sheltered sites was discernible in the second size class corresponding to a shell length of 5-6mm and an age of 5-6mo, becoming more pronounced in successive size classes (Fig. S4).

**Experiment 2: wave-exposure gradient**

To represent a greater range of exposure to wave action, thirty adults were collected in March 2002 from the shores used in experiment 1 plus two extra shores, Friars Bay and Caethle (Fig. S3). Each population was housed in a separate aquarium and fed ad lib on mussels until sufficient numbers of egg capsules had been laid. Egg capsules were transferred to 2 l plastic bottles filled with gently aerated seawater held at 15°C. Two bottles were assigned to each population. Mussels <10mm shell length were provided ad lib as food. Snails were measured at 12 months, when they had grown to a shell length ≥16mm and some had begun to lay eggs. Samples of 20-35 snails were available per population. Log_10_-transformed data were subjected to ANCOVA; after confirming non-heterogeneity of slopes, Bonferroni-corrected paired comparisons were made of mean aperture width adjusted to shell length.

Relative aperture external width of laboratory-reared snails was correlated with that of their ancestral field populations (Fig. S5; Pearson r = 0.893, P = 0.017), indicating a heritable component of variation in shell morphology. Relative aperture external width was also positively ranked with wave exposure at the study sites (Fig. S5; laboratory snails, Spearman rho = 1.000; field snails, Spearman rho = 0.943, P = 0.005).

Because of the relatively small range of shell length (5mm) per group, log transformation was not used.
